# The roles of bone‐derived exosomes and exosomal microRNAs in regulating bone remodelling

**DOI:** 10.1111/jcmm.13039

**Published:** 2016-11-23

**Authors:** Yong Xie, Yanyu Chen, Licheng Zhang, Wei Ge, Peifu Tang

**Affiliations:** ^1^ Department of OrthopedicsChinese PLA General HospitalBeijingChina; ^2^ National Key Laboratory of Medical Molecular Biology and Department of ImmunologyInstitute of Basic Medical SciencesChinese Academy of Medical SciencesBeijingChina

**Keywords:** exosome, microRNA, osteoclast, osteoblast, bone remodelling

## Abstract

Pathological destructive bone diseases are primarily caused by the failure of a lifelong self‐renewal process of the skeletal system called bone remodelling. The mechanisms underlying this process include enhanced osteoclast activity and decreased generation of the osteoblast lineage. Intercellular interaction and crosstalk among these cell types are crucial for the maintenance of bone remodelling, either through the secretion of growth factors or direct cell–cell physical engagement. Recent studies have revealed that exosomes derived from bone cells, including osteoclasts, osteoblasts and their precursors, play pivotal roles on bone remodelling by transferring biologically active molecules to target cells, especially in the processes of osteoclast and osteoblast differentiation. Here, we review the contents of bone‐derived exosomes and their functions in the regulatory processes of differentiation and communication of osteoclasts and osteoblasts. In addition, we highlight the characteristics of microRNAs of bone‐derived exosomes involved in the regulation of bone remodelling, as well as the potential clinical applications of bone‐derived exosomes in bone remodelling disorders.

## Introduction

Pathological destructive bone diseases 1, including osteoporosis, osteoarthritis (OA) and rheumatoid arthritis (RA) 2, are associated with a persistent decrease in the patient's quality of life 3 and are also considered to present a major global health problem 4. These disorders are primarily caused by the failure of bone remodelling processes, including enhanced osteoclast activity or decreased generation of the osteoblast lineage 1.

Bone remodelling, which takes place in the bone remodelling compartment, is a continuous and lifelong process of repair of micro‐damage to the bone structure and the replacement of ageing bone tissue 5. It is a prerequisite for the maintenance of bone mass and the mechanical strength of bone 6. In bone remodelling, the destructive process that involves resorption of bone by osteoclasts is coupled with a productive process, in which bone is synthesized by osteoblasts 7. Osteoclasts, which share precursors with macrophages, are derived from haematopoietic stem cells (HSCs) 8 and are unique in their function of the resorption of bone matrices 9. In contrast, along with adipocytes and chondrocytes, osteoblasts and their constituent progenitor cells originate from mesenchymal stem cells (MSCs) 10. The coordinated regulation of osteoblasts and osteoclasts, which is critical for maintaining physiological bone remodelling 11, is tightly controlled by modulating molecular signals 12. A number of studies have focused on the specific factors involved in bone remodelling to identify new therapeutic strategies for bone disorders 13, 14. The receptor activator of nuclear factor‐κB ligand (RANKL) and macrophage colony‐stimulating factor (M‐CSF) 15 activate various intracellular signalling pathways to regulate the transcription and expression of osteoclast‐specific genes 16. Members of the transforming growth factor‐beta (TGF‐β) families are essential for osteoblast formation 17. In this process, communication between osteoclasts and osteoblasts occurs through the secretion of regulatory factors 18, or *via* direct physical interactions, such as the engagement of ephrin/Eph receptors 19. Additionally, recent studies have reported that key factors involved in bone remodelling are packaged in spherical bilayered membrane vesicles called exosomes 20. These organelles function as cell–cell communicators by transferring biologically active molecules to adjacent or distant cells 21.

Various types of cells, such as epithelial and haematopoietic cells, secrete exosomes. The latest studies have demonstrated that bone‐related cells, including osteoclast precursors, osteoclasts, MSCs, osteoblasts and osteocytes, also secrete exosomes 22. These small vesicles (average diameter 40–100 nm) are derived from endosomal membranes after the fusion of multivesicular bodies to the plasma membrane. Cells can release exosomes from the plasma membrane by outward budding, which is a calcium‐dependent process 23. Exosomes contain numerous bioactive molecules, which vary according to the specific donor cell type. Generally, exosomes are enriched with protein members of the transmembrane 4 superfamily (CD9, CD63 and CD81) and tumour susceptibility gene 101 (TSG101) 24. Although the removal of unnecessary proteins from parent cells is known to be the primary function of exosomes, their functional characteristics are not completely clear 25. Exosomes transfer their luminal components, including proteins, microRNAs (miRNAs) and enzymes, to their target cells 26. It is therefore likely that rather than simply acting as vessels for the removal of cellular debris, exosomes function as extracellular organelles with paracrine/endocrine roles in intercellular communication 27.

Accumulating evidence supports the endocrine function of the skeleton 28. The bone matrix is produced by mature osteoblasts, which are generated from osteoblast precursors. Osteocytes, which are terminally differentiated cells of the osteoblast lineage, are embedded in the mineralized extracellular matrix and are involved in the regulation of bone remodelling 29. Osteoblasts and osteocytes respond to mechanical stimuli by secreting paracrine/autocrine factors that maintain bone mass 30 through the renewal and differentiation of precursors from the bone marrow (BM) progenitor pool, as well as bone formation and resorption 31. These processes occur in the bone matrix canaliculi, through which nutrients and oxygen pass from blood vessels to bone cells and signalling molecules are transported intercellularly, allowing communication between cells deep within the bone matrix and those at the surface without direct contact 29. Recent reports indicate the involvement of bone‐derived exosomes in regulating bone remodelling, mainly *via* the transfer of critical molecules required for the differentiation and communication of osteoclasts and osteoblasts 32.

The contents of exosomes vary according to their origin. To provide a better understanding of the mechanisms by which bone‐derived exosomes regulate bone remodelling, we have reviewed the latest discoveries regarding the changing characteristics and roles of bone‐derived exosomes during the regulation of osteoclast and osteoblast differentiation. Furthermore, we highlight the characteristics of bone‐derived exosomal microRNAs involved in regulation of bone remodelling as well as the potential clinical applications of bone‐derived exosomes in bone remodelling disorders.

## The characteristics and contents of bone‐derived exosomes

Although the processes by which bioactive molecules are packaged into exosomes are largely unknown, recent reports suggest the existence of a specific and tightly controlled mechanism 33. Isolation of exosomes from mature osteoclasts and precursors revealed they had similar sizes and morphology, as well as expression of specific markers, including epithelial cell adhesion molecule 34 and CD63 35. Sun *et al*. 32 indicated that ephrinA2 protein was enriched in osteoclast‐derived exosomes. Furthermore, ephrinA2 levels in the serum of osteoporotic mice and patients were found to be significantly up‐regulated. These results suggested that osteoclast‐derived exosomal ephrinA2 is a marker for recognition with osteoblasts, and the engagement of ephrinA2/EphA2 is essential for exosome‐mediated communication between osteoclasts and osteoblasts. Receptor activator of nuclear factor κB (RANK) was detected at low levels in the exosomes from precursors but at much higher levels in mature osteoclasts 20. In accordance with the induction of RANKL by bone resorption factors, such as parathyroid hormone (PTH), RANKL expression increased significantly in exosomes secreted from PTH‐treated osteoblasts 22. In addition, PTH caused osteoblasts to release exosomes containing osteoblast membrane proteins and the exosome marker flotillin‐2 36. Proteomic analysis of osteoblast‐derived exosomes 37 revealed that the exosomal proteins are predominantly involved in protein localization and intracellular signalling cascade and are mainly located in the plasma membrane and cytosol, with molecular functions focused on nucleotide binding and structural molecule activity. Furthermore, exosomal proteins were enriched among the eukaryotic initiation factor (EIF)2, protein ubiquitination and integrin signalling pathways. In total, 23 proteins, including EIF family members, PP1C and PABP, were the mapped to EIF2 signalling pathway. Osteoblasts were derived from MSCs, thus implicating putative roles of MSC‐derived exosomes in the regulation of bone remodelling. Lai *et al*. 38 identified 857 exosomal proteins within MSC‐derived embryonic stem cell lines. Mesenchymal stem cells‐derived exosomes expressed the characteristic markers, CD13, CD29, CD44, CD73 and CD105 39. Among the 1069 proteins identified in exosomes isolated from the MSC‐derived osteoblast precursor MC3T3 cell line, 786 proteins are present in the ExoCarta database 37. Seven messenger RNA (mRNAs), ACIN1, DDX6, DGKA, DKK2, Lsm2, RPS2 and Xsox17, showed significant differential expression over time in exosomes from differentiated human bone marrow‐derived mesenchymal stem cells (HBMSCs), which can be induced to differentiate into mineralized osteoblasts. Furthermore, dysregulated exosomal expression of two NF‐κB‐related genes, ADAM17 and NF‐κB1, was detected in osteogenic differentiated HBMSCs 40. Differential expression of some miRNAs was also detected in the exosomes of mineralizing osteoblasts and HBMSCs, including some that have been confirmed to be functionally associated with bone remodelling 40, 41.

## The roles of bone‐derived exosomes in bone remodelling

Similar to the function of cytokines and soluble factors, bone‐derived exosomes can recruit BM‐derived cells, such as HSCs (precursors of osteoclasts) and MSCs (precursors of osteoblasts), to the bone surface 25. Osteoclast precursor‐derived exosomes stimulated the formation of significantly greater numbers of osteoclasts compared with the numbers formed in the absence of exosomes. In contrast, significantly fewer osteoclasts were formed in the presence of osteoclast‐derived exosomes compared with the numbers determined in 1,25‐(OH)2D3‐stimulated mouse marrow. RANK levels were much higher in osteoclast‐derived exosomes, and the removal of exosomes containing RANK significantly alleviated the inhibition of osteoclastogenesis 20. Therefore, RANK‐rich exosomes may function as novel inhibitors by binding competitively to RANKL, thus preventing stimulation of the RANK signalling pathway in osteoclasts 42. Furthermore, there was no significant change in differentiation of mouse BM haematopoietic precursors stimulated with recombinant RANKL and M‐CSF to mature osteoclasts following their exposure to exosomes isolated from osteoclast precursors and osteoclasts 20. These data imply that the inhibitory function of RANK‐rich exosomes on osteoclastogenesis may be alleviated in the presence of high levels of RANKL.

**Figure 1:**
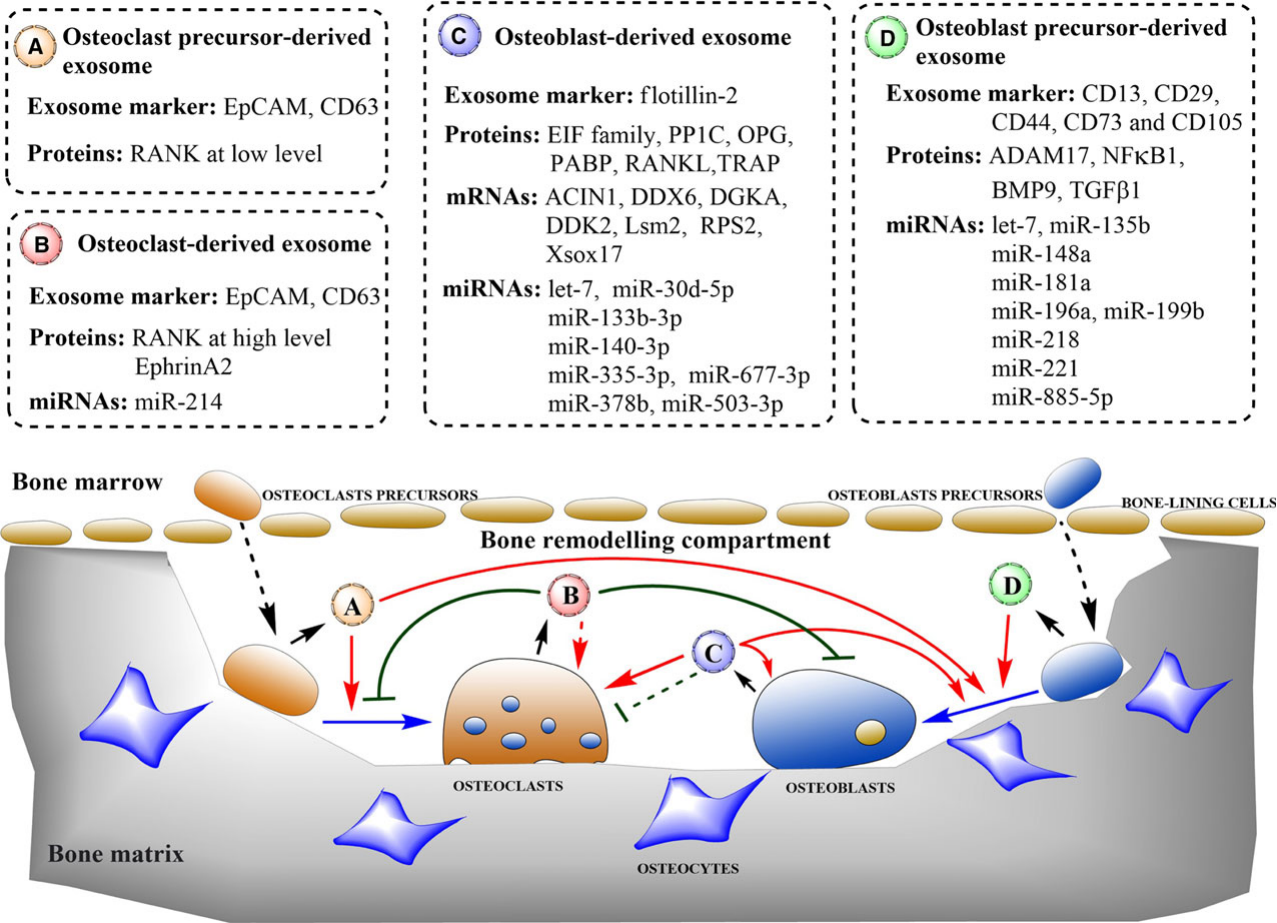
Schematic diagram showing roles of bone‐derived exosomes in regulating bone remodelling. Bone‐derived exosomes can regulate differentiation of osteoclasts and osteoblasts by transferring biologically active molecules to target cells. Osteoclast precursor‐derived exosomes (A) stimulate the differentiation of osteoclasts and osteoblasts. Osteoclast‐derived exosomes (B) reduce the number of osteoclasts formed and osteoblastic bone formation. Furthermore, osteoclast‐derived exosomes containing miR‐214 may promote the osteoclast differentiation. Osteoblast‐derived exosomes (C) promote differentiation of osteoblasts and osteoclasts, as well as establish a positive feedback in bone growth. In addition, osteoblast‐derived exosomes containing OPG may inhibit the osteoclast differentiation. Osteoblast precursor‐derived exosomes (D) induce MSCs to differentiate into osteoblasts. Dotted boxes indicate the primary contents of bone‐derived exosomes that are involved in bone remodelling. Short black arrows indicate the secretion process. Dotted black arrows indicate the translocation of cells. Solid blue arrows indicate the differentiation process. Solid red arrows indicate the promotion of cellular processes, and solid green lines indicate inhibition of cellular processes. Dotted lines indicate that the mechanism has not been fully elucidated.

The roles of exosomes in osteoclasts may provide clues as to how bone formation and absorption are orchestrated 20. Exosomes from BM stromal cells (BMSCs) can also be involved in bone remodelling by directly regulating osteoblast proliferation and activity 43. Mesenchymal stem cells ‐derived exosomes have been shown to up‐regulate expression of the growth factors, bone morphogenetic protein 9 (BMP9) and transforming growth factor‐β1(TGF‐β1) 44, both of which effectively induce the osteogenic differentiation of MSCs 45. BMP9 induces osteogenic differentiation with greater potency than BMP2 46. Mesenchymal stem cells ‐derived exosomes bind and tether extracellular matrix (ECM) proteins, such as type I collagen and fibronectin, to the bone surface and biomaterials. This function also allows the use of MSC‐derived exosomes as biomimetic tools that induce the differentiation of BMSCs into an osteogenic lineage 47. In addition, exosomes are released by the osteoblast itself, thus establishing a positive feedback mechanism that promotes bone growth. Exosomes from the mineralizing MC3T3‐E1 (mature osteoblast cell line) promoted osteogenic differentiation of the ST2 osteoblast precursor cell line, manifested by the up‐regulated expression of osteogenic markers, runt‐related transcription factor 2 (RUNX2) and alkaline phosphatase, as well as enhanced matrix mineralization 41. Furthermore, EIF2 in osteoblast‐derived exosomes may also induce MSCs to differentiate into osteoblasts 37. Bone remodelling is known to be closely regulated by the interaction between osteoblasts and osteoclasts. In fact, osteoblast‐ and osteocyte‐derived lysosomal membrane protein 1 (LAMP1)‐positive exosomes also contain tartrate‐resistant acid phosphatase, RANKL and osteoprotegerin (OPG), which are critical to osteoclast differentiation 48. As Deng *et al*. 22 identified, RANKL‐rich exosomes released from osteoblasts stimulated osteoclast formation. In addition, Omar *et al*. and Ekstrom *et al*. indicated that exosomes secreted from monocytes, which are derived from HSCs and share precursors with osteoclasts, stimulated the osteogenic differentiation of MSCs 49, 50. The recent studies of Li *et al*. and Sun *et al*. demonstrated a novel models of inhibitory effect on osteogenic differentiation by osteoclast‐derived miR‐214‐containing exosomes 32, 51. Although the differentiation of osteoclasts and osteoblasts is under the regulation of bone‐derived exosomes (Fig. 1), the cell type from which the most potent regulatory exosomes are derived and the mechanism by which the exosomes mediate bone remodelling remain to be investigated 42.

Matrix vesicles (MVs), known to be important in the development of vertebrate mineralizing tissue, share structures that are homologous to those that anchor exosomes to the ECM as well as similarities in morphological appearance and functional activities 52. Scanning electron microscope (SEM) observations showed that MVs are either scattered among collagen fibres, or aggregated on the cell surface 52. Recent studies suggest that autophagosomes containing mineral nuclei are released from the cell and transported to the plasma membrane as mineralizing exosomes 53. This process implicates autophagic activity in endosomal processing and indicates that mineralization starts within vacuoles before export from the cell. Thus, mineralization, nucleation and crystal growth are likely to be regulated by bone‐derived exosomes 52.

## The involvement of bone‐derived exosomal miRNAs in regulating bone remodelling

The function of exosomal miRNAs has become a focus of research because of their pivotal role in gene expression regulation. microRNAs are small endogenous non‐coding RNA molecules, containing approximately 22 nucleotides. They are well known as post‐transcriptional regulators of messenger RNA (mRNA) expression 54. Within the cytoplasm, the enzyme known as Dicer converts pre‐miRNA into miRNA 55, which is loaded into the RNA‐induced silencing complex (RISC) 56. The RISC binds to the seed region of the target mRNA 57. Although the binding complementarity is usually imperfect, silencing of target mRNA by the associated miRNAs impacts on between 1% and 4% of human gene expression 58. After packaging into exosomes, miRNAs and the RISC are transported through the interstitium or even to the peripheral blood 59. Exosomal miRNAs are more resistant to ubiquitous RNases, extreme temperatures and pH levels, and prolonged storage in the extracellular environment. In addition, plasma membrane fusion and endocytosis are the currently identified mechanisms that account for the uptake of exosomal miRNAs by their target cells, where they exert their biological functions 59.

Recent research has revealed the involvement of a number of bone‐derived exosomal miRNAs in the regulation of bone remodelling (Table 1). Forty‐three miRNAs were highly expressed in mineralized MC3T3‐E1 exosomes, including miR‐30d‐5p, miR‐133b‐3p and miR‐140‐3p, which were functionally confirmed to be involved in regulating remodelling of bone tissues. These miRNAs may participate in multiple pathways that are important in osteoblast differentiation and function, such as the Wnt, insulin, TGF‐β and calcium signalling pathways 41. Nine miRNAs (let‐7a, miR‐135b, miR‐148a, miR‐199b, miR‐203, miR‐218, miR‐219, miR‐299‐5p and miR‐302b) were up‐regulated in HBMSC exosomes during osteogenic differentiation. In contrast, five miRNAs (miR‐155, miR‐181a, miR‐221, miR‐320c and miR‐885‐5p) were significantly down‐regulated in exosomal samples. These down‐regulated miRNAs and their cotarget genes are enriched in the insulin signalling pathway and also the mitogen‐activated protein kinase and phosphoinositide 3‐kinase/Akt pathways, both of which play pivotal roles in osteoblast differentiation 40.

**Table 1:** Bone‐derived exosomal miRNAs involved in regulating bone remodelling

microRNA	miRNA expression	Target gene	Cell line	miRNA function	Reference
miR‐30d‐5p	↑	*RUNX2*	Mineralizing MC3T3‐E1	Inhibits osteoblast differentiation	62
miR‐133b‐3p	↑	*RUNX2*	Mineralizing MC3T3‐E1	Inhibits osteoblast differentiation	63
miR‐140‐3p	↑	*BMP2*	Mineralizing MC3T3‐E1	Inhibits osteoblast differentiation	69
let‐7	↑	*AXIN2* *HMGA2*	HBMSCs Mineralizing MC3T3‐E1	Enhances osteoblast differentiation	60, 61
miR‐135b	↑	*CADM3* *GPR12* *COL15A1*	HBMSCs	Involves in the impaired osteogenic differentiation	66, 67
miR‐148a	↑	*MAFB*	HBMSCs	Promotes osteoclastogenesis	76, 77
miR‐199b	↑	*RUNX2*	HBMSCs	Possibly involved in the control of osteoblast differentiation	65
miR‐221	↓	*RUNX2*	HBMSCs	Inhibits osteoblast differentiation	64
miR‐218	↑	*SOST* *DKK2 SFRP2* *DKK2 SFRP2*	HBMSCs	Enhances osteoblast differentiation	70
miR‐196a	↑	*HOXC8*	BMSCs	Enhances osteoblast differentiation	42, 71
miR‐181a	↓	*TGF‐BI* *T*β*R‐I* *RGS4* *GATA6*	HBMSCs	Enhances osteoblast differentiation	72
miR‐335‐3p	↑	*DKK1*	Mineralizing MC3T3‐E1	Enhances osteoblast differentiation	73
miR‐378b	↑	*CASP3*	Mineralizing MC3T3‐E1	Enhances osteoblast differentiation	74
miR‐503‐3p	↑	*RANK*	Mineralizing MC3T3‐E1	Inhibits RANKL‐induced osteoclast differentiation	75
miR‐885‐5p	↓	*RUNX2*	HBMSCs	Inhibits osteoblast differentiation	40
miR‐677‐3p	↑	*AXIN1*	Mineralizing MC3T3‐E1	Promotes MSC osteogenic differentiation	41
miR‐214	↑	*ATF4* *PTEN*	Osteoclasts	Inhibits osteoblastic bone formation Might promote osteoclastogenesis	32, 51, 79

In addition, osteoblastic differentiation‐related miRNAs are greatly increased in mineralizing osteogenic ST2 cells, with 91 up‐regulated and 182 down‐regulated after coculture with mineralized osteoblast‐derived exosomes. Among the 91 overexpressed miRNAs, 18 were contained in mineralized osteoblast‐derived exosomes, including four highly expressed miRNAs (miR‐677‐3p, miR‐680, miR‐3084‐3p and miR‐5100). Only 20% of miRNAs overexpressed in recipient cells were detected in exosomes from original osteoblasts, suggesting that the transfer of exosomal miRNAs only contributes partly to the altered miRNA expression in recipient cells, and other mechanisms that modulate the miRNA expression of recipient cells might be existed. Among the miRNAs overexpressed in recipient cells, five up‐regulated miRNAs (miR‐667‐3p, miR‐874‐3p, miR‐6769b‐5p, miR‐7044‐5p and miR‐7668‐3p) cotarget AXIN1, which is a key β‐catenin inhibitor. β‐catenin is an essential transcription factor involved in osteoblast differentiation *via* the Wnt signalling pathway. AXIN1 expression was repressed and β‐catenin expression was enhanced after transfer of mineralized osteoblast exosomes, thereby promoting the osteogenic differentiation of precursors of osteoblasts 41.

Let‐7, which is found in exosomes from both mineralized osteoblasts and osteoblast precursors, was shown to enhance osteogenesis by regulating high‐mobility group AT‐hook 2 (HMGA2) and AXIN2 60, 61. A numbers of bone‐derived exosomal miRNAs, such as miR‐30d‐5p, miR‐133b‐3p, miR‐199b, miR‐221 and miR‐885‐5p, have been reported to be involved in the control of osteoblast differentiation by RUNX2. miR‐30d‐5p and miR‐133b‐3p, which are known to inhibit osteoblast differentiation by targeting the *RUNX2* gene, were highly expressed in osteoblast‐derived exosomes 62, 63. In contrast, miR‐221 and miR‐885‐5p, which function as negative regulators of osteogenic differentiation by repressing RUNX2, were down‐regulated in exosomes from HBMSCs 40, 64. miR‐199b was also found to be involved in the control of RUNX2‐mediated osteoblast differentiation 65. Mineralization during osteogenic differentiation of human unrestricted somatic stem cells is regulated by miR‐135b 66, which is also involved in impaired osteogenic differentiation of MSCs 67. Wnt5a, a classical non‐canonical Wnt, was recently reported as a critical component of BMP2‐mediated osteogenic differentiation 68. miR‐140‐3p, which is highly expressed in osteoblast‐derived exosomes, inhibits osteoblast formation by repressing BMP2 expression 69. However, miR‐218/Wnt signalling promotes osteoblast differentiation and activity by repressing sclerostin, an inhibitor of osteoblast formation released by osteocytes 70. miR‐196a is the key factor involved in stimulating the proliferation and activity of osteoblasts 42, 71. Both miR‐196a and miR‐218 were up‐regulated in MSC‐derived exosomes. However, miR‐181a, a positive regulator for osteoblast differentiation 72, was down‐regulated in HBMSCs. Furthermore, miR‐335‐3p, miR‐378b and miR‐677‐3p, which were up‐regulated in mineralizing MC3T3‐E1 cells, are associated with enhanced osteoblast differentiation through the repression of their target genes 73, 74.

As communicators in bone remodelling, exosomes derived from the osteoblast lineage contain miRNAs that target key osteoclast differentiation factors. miR‐503‐3p from mineralized osteoblast‐derived exosomes may inhibit RANKL‐induced osteoclast differentiation by regulating RANK expression 75. In contrast, miR‐148a, which is known to promote osteoclastogenesis by targeting the *MAFB* gene 76, 77, was found to be up‐regulated in HBMSC exosomes. In addition, Li, *et al*. showed that elevated serum exosomal miR‐214‐3p is associated with reduced bone formation in both elderly women and ovariectomized (OVX) mice 51. Serum exosomal miR‐214 levels were then found to be significantly elevated in osteoclast‐specific miR‐214 transgenic mice. The miR‐214 enriched in osteoclast‐derived exosomes can be transferred into osteoblasts to inhibit their activity *via* ephrinA2/EphA2 32. A previous study by Wang *et al*. 78 identified that miR‐214 inhibits osteoblast function by targeting *ATF4*, while their further experiments identified that miR‐214 promotes osteoclastogenesis through PI3K/Akt pathway by targeting the Pten tensin homologue 79. Therefore, miR‐214‐containing exosomes from osteoclasts may have multiple roles that favour bone destruction. Future investigations are required to clarify the potential functions of these exosome‐associated miRNAs in regulating bone remodelling *via* mechanisms such as paracrine/autocrine signalling or in communication between osteoclasts and osteoblasts or with other cell types 40.

## Conclusions and perspective

Here, a new aspect of the roles of bone‐derived exosomes in bone remodelling has been elucidated. The content of bone‐derived exosomes, including the proteins, mRNAs and miRNAs, varies according to different parent cells. Therefore, bone‐derived exosomes exert multiple roles in bone remodelling (Fig. 1). Exosomes originating from osteoclast precursors activate differentiation of osteoclasts and osteoblasts. In contrast, RANK‐containing exosomes from osteoclasts inhibit osteoclast formation. Osteoclast‐derived exosomes also inhibit osteoblast formation or might promote osteoclastogenesis in terms of the high expression of miR‐214. Furthermore, exosomes isolated from osteoblast precursors promote the formation and activities of osteoblasts, while exosomes derived from mature osteoblasts either induce the differentiation of MSCs into mineralizing osteoblasts and osteocytes or establish positive feedback among osteoblasts themselves. In addition, osteoblast‐derived exosomes containing OPG may inhibit the osteoclast differentiation. Thus, osteoblast‐derived exosomes may exert antagonistic or synergistic activities on osteoclasts through transporting their contents. We can imply and then infer that the exosomes from mineralizing osteoblasts, as well as precursors of osteoclasts and osteoblasts are principally involved in promoting bone remodelling by facilitating either osteogenesis or osteoclastogenesis. However, the mature osteoclast‐derived exosomes are likely to be inhibitory factors for bone remodelling through alleviating osteoclast differentiation and osteoblast formation.

Bone‐derived exosomal miRNAs are thought to be important in regulating gene expression involved in the differentiation and communication between multiple cell types responsible for bone formation and resorption. Recent studies have shown that Wnt5a enhances osteoclast formation through β‐catenin‐dependent signalling 80. RUNX2 is a key transcription factor that regulates osteogenesis, while osteocytes secrete sclerostin (encoded by the *SOST* gene) to inhibit the activity of osteoclasts and osteoblasts 81. According to the literature, many of the key factors that regulate osteoclasts and osteoblasts are targeted by the different miRNAs contained within specific bone‐derived exosomes, such as RUNX2, BMPs and sclerostin 42. However, exosomal miRNAs from the same parent cells may have opposing functions in terms of osteoclast differentiation and osteoblast activity. Thus, this implies that bone‐derived exosomes may not perform functions such as cell–cell interaction during the regulation of bone remodelling that are completely in accordance with those of the parent cells. microRNAs alterations in the recipient do not match the abundance of miRNA in the donor exosomes 41, suggesting that components of osteoblast‐derived exosomes other than miRNAs may also alter the miRNA profile of recipient cells 41. The endosomal sorting complexes required for transport (ESCRT) machinery functions as a link between miRNAs and exosomes. In this system, the RISC acts together with the ESCRT complex to load miRNAs into exosomes 59. miRNAs form an integral part of the RISC complex, which mediates the efficient transfer of the miRNA to its target in the recipient cell. Furthermore, exosomes incorporate precursor miRNAs (pre‐miRNAs) in complexes with Dicer, TRBP and AGO2 proteins, to facilitate their processing in a cell‐independent manner 82.

The cargo of tumour‐derived exosomes vary according to the cancer types and tumour characteristics 83, 84, and are more relevant in malignancy than benign bone diseases. Therefore, information on the crosstalk between cancer cells and the bone microenvironment through exosomes is not discussed in the present paper, even though it is important to consider its effect on bone homeostasis. In terms of the multiple and complex interactions between types of bone cells reviewed herein, further investigations are needed to fully elucidate the contents and potential modulatory roles of bone‐derived exosomes in regulating bone remodelling, especially the comprehensive protein and miRNA contents of osteoclast‐derived exosomes as well as which types of bone‐derived exosomes play dominant roles in the regulation of bone remodelling and under what circumstances they would be activated. Encapsulation by the lipid bilayer of the exosomal membrane protects proteins and miRNAs from degradation 85 and they can be assayed in small peripheral blood samples 33. Thus, the different expression level of specific contents of bone‐derived exosomes might serve as a promising diagnostic and/or prognostic tool to detect early‐stage bone disorders. Furthermore, engineered exosomes represent a promising system that can be used for the targeted delivery of RNAi molecules, while avoiding detection by the immune system 86. In this way, the transfer of particular ESCRT machinery or miRNAs might facilitate the treatment of bone diseases that are related to aberrant bone remodelling, such as RA, OA and osteoporosis.

## Conflict of interest

All co‐authors implicated in this review declare that they have no conflict of interest.
